# Internal Nasal Valve Collapse Treatment by Endonasal Hyaluronic Acid Injection

**DOI:** 10.1007/s00266-024-04186-9

**Published:** 2024-09-12

**Authors:** Pierre Gagnieur, Maxime Fieux, Laurie Saloner, Bruno Louis, Delphine Vertu-Ciolino, Alain-Ali Mojallal

**Affiliations:** 1 Service de chirurgie maxillo-faciale et plastique de la face, Hospices Civils de Lyon, Centre Hospitalier Lyon Sud, F-69495 Pierre Bénite cedex, France; 2 Service d’ORL d’otoneurochirurgie et de chirurgie cervico-faciale, Hospices Civils de Lyon, Centre Hospitalier Lyon Sud, F-69495 Pierre Bénite cedex, France; 3 Université de Lyon, Université Lyon 1, F-69003 Lyon, France; 4 Univ Paris Est Créteil, INSERM, F-94010 Créteil, IMRB France; 5 CNRS, EMR 7000, F-94010 Créteil, France; 6 Service d’ORL et de chirurgie cervico-faciale, Hospices Civils de Lyon, Hôpital Edouard Herriot, F-69003 Lyon, France; 7 UMR 5305, CNRS, LBTI, F-69007 Lyon, France; 8 Service de Chirurgie Plastique, Esthétique et Réparatrice, Hospices Civils de Lyon, Hôpital Croix Rousse, F-69003 Lyon, France

**Keywords:** Nasal airflows dynamics, Quality of life, Therapeutics, Post-operative

## Abstract

**Objective:**

Internal nasal valve collapse (IVC) is a common functional complication of rhinoplasty and injecting hyaluronic acid is one of the treatment options available, but its effectiveness has never been evaluated. The objective of this study was to assess the evolution of IVC after injection of hyaluronic acid using objective and subjective measures of nasal obstruction.

**Study Design:**

A prospective interventional study was conducted.

**Methods:**

Adult patients consulting for nasal obstruction after (septo)rhinoplasty and diagnosed with IVC were included. Patients underwent 4-phase rhinomanometry, completed nasal obstruction symptoms evaluation (NOSE) and visual analog scale (VAS) questionnaires and received hyaluronic acid injections. Measurements were repeated immediately, one month and one year later. The primary outcome measure was the proportion of patients below the rhinomanometric diagnostic threshold for IVC at one month.

**Results:**

Among the 22 patients included, 20 (91%) had rhinomanometry measurements below the diagnostic threshold for IVC one month after injection. It decreased to 53% (8/15 patients) at one year post injection. The mean NOSE score decreased from 74.5 (± 18.0) before injection to 35.2 (± 23.3) after injection (p < 0.0001). The mean VAS score decreased from 7.0 (± 1.4) before injection to 3.4 (± 1.9) after injection (p < 0.0001). In these patients with post-(septo)rhinoplasty IVC, hyaluronic acid injection into the internal nasal valve substantially improved subjective and objective measures of nasal obstruction.

**Conclusion:**

These results suggest hyaluronic acid injection (performed as described) is an effective treatment for IVC and is an excellent alternative to surgical treatment.

**Level of Evidence III:**

This journal requires that authors assign a level of evidence to each article. For a full description of these Evidence-Based Medicine ratings, please refer to the Table of Contents or the online Instructions to Authors https://www.springer.com/00266.

**Supplementary Information:**

The online version contains supplementary material available at 10.1007/s00266-024-04186-9.

## Introduction

The internal valve region, bounded by the septum, the caudal edge of the upper lateral cartilage (ULC) and the cephalic edge of the lower lateral cartilage, is the flow-limiting segment of the nasal cavity (1). On entering this constricted segment, the airflow accelerates, leading to a drop in the intraluminal pressure according to Poiseuille’s law (2). Depending on the rigidity of the cartilaginous and ligamentous structures (3), this pressure drop can lead to internal nasal valve collapse (IVC, i.e. the ULC collapses to the septum), resulting in nasal obstruction. Internal nasal valve collapse is diagnosed clinically (4) by observing the collapse of the ULC during light or moderate inspiration, and/or by positivity of the modified Cottle maneuver [breathing facilitated by passive abduction of the ULC using a cotton swab (5)]. Internal valve collapse can also be diagnosed objectively by 4-phase rhinomanometry (1, 6).

Nearly 80% of cases of IVC are subsequent to rhinoplasty (7, 8). This is because of the increasing popularity of rhinoplasty (215,000 procedures performed in 2017 in the US, the 3^rd^ most frequent cosmetic surgery procedure^9^) and the high prevalence of postoperative functional problems [thought to affect 10% of patients (9, 10)], among which IVC is the most common (11). Preventing IVC is crucial during primary surgery. This includes sparing the scroll area (12) and reconstructing the middle third after nasal hump reduction (13, 14). Treatment is mainly surgical (5, 15–18), through secondary rhinoplasty, but the operation is intricate and time-consuming (19). As an alternative to surgery, Nyte et al. proposed replacing cartilage spreader grafts with hyaluronic acid (HA) injections into the internal nasal valve (20). The advantages of this procedure are that it is quick, inexpensive, (21) can be performed in everyday clinical practice, does not require general anesthesia, and offers immediate improvements, with simple postoperative follow-up, and low complication rates (22, 23).

While, this technique has been known since 2007(20), and is used routinely (24), to our knowledge, no data on its effectiveness have ever been published. We therefore evaluated the efficacy of HA injection into the internal nasal valve (along the ULC-septum angle) of patients with post-rhinoplasty IVC. The primary outcome measure was the proportion of patients without IVC one month after injection, as assessed objectively by rhinomanometry. Secondary outcomes of interest were the associated changes in the nasal obstruction symptoms evaluation (NOSE) scale (25, 26) and the nasal obstruction visual analog scale (VAS) (27, 28).

## Material and Methods

### Study Design

This was a prospective interventional study of all adult patients (>18 years) seen in a tertiary referral center between January and April 2023 for nasal obstruction due to IVC following (septo)rhinoplasty. Nasal obstruction due to IVC was defined as the combination of i) perceived nasal obstruction (uni- or bilateral), ii) ULC collapse during normal nasal inspiration, iii) alleviation of nasal obstruction by passive abduction of the ULC with a cotton swab (positive modified Cottle maneuver), and iv) an inspiratory loop area greater than 17.3 Pa·L· s^-1^ in the 4-phase rhinometry flow/pressure diagram (6).

Patients with other causes of nasal obstruction (residual septal deviation, mucosal synechiae, septal perforation, turbinate hypertrophy, collapsed external valve, etc.) or with a contraindication to HA injection were excluded. The primary variable of interest was the hysteresis loop area (measured during the inspiratory phase on 4-phase rhinomanometry flow/pressure diagrams), as measured before injection (D0), 30 days after injection (D30) and twelvemonths after injection (M12). The secondary variables of interest were the patients’ NOSE scores (before injection, on D30 and M12) and nasal obstruction VAS scores (before injection, 5 min after, on D30 and M12).

### Ethic

The study was conducted in accordance with the Declaration of Helsinki and was approved by the *Comité de Protection des Personnes* Paris - Ile de France Hôpital Saint Louis on 25 November 2021 (ID 2021-A02509-32). The trial was registered at ClinicalTrials.gov (NCT05134831). All data were anonymized. Written informed consent was obtained from all participants and patients signed a consent form for the use of their photographs.

### Collected Data

Patients were evaluated at the inclusion visit (D0) and at the two follow-up visits (D30 and M12). The data collected at the inclusion visit (D0) were the patients’ age, sex, body mass index (BMI), atopic status, smoking status, and history of nasal trauma and nasal or sinus surgery. The patients received a physical examination and 4-phase rhinomanometry measurements were performed. The physical examination included i) a static examination of the nasal pyramid: shape (Caucasian, African, Asian), presence of deviation, presence of an “inverted V” deformity, width of the middle third (thin, normal, wide), profile appearance of the dorsum (straight, hump, kyphosis), presence of septal deviation and/or inferior turbinate hypertrophy; and ii) a functional examination: observation of ULC collapse on weak or moderate inspiration, effect of passive abduction of the lateral cartilage with a cotton swap (modified Cottle maneuver), and effect of lateral traction of the nostril and cheek (Cottle maneuver). The 4-phase rhinometry measurements were performed by a senior surgeon using a Rhinolab 4-Rhino device (Rhinolab GmbH, Freiburg, Germany) and the 4-Rhino software (v. 6.1.1). The patients were seated and had rested for 30 min beforehand. The contralateral nostril was occluded with medical tape, to avoid modifying the structure of the nasal wing and the nasal valve area. The data collected for each nasal cavity were the log of the vertex resistance (VR), the log of the effective resistance (ER), and flow/pressure curves. The hysteresis area in these curves was calculated (6) separately for each nasal cavity. Patients then completed the NOSE nasal obstruction VAS questionnaires (both presented in the Appendix).

At the two follow-up visits after injection (D30 and M12), patients completed the NOSE and nasal obstruction VAS questionnaires, and their nasal breathing was assessed by 4-phase rhinomanometry as described above.

### Hyaluronic Acid Injections

Hyaluronic acid injections were performed at the end of the inclusion visit. The nasal vestibule was meticulously swabbed bilaterally with non-woven gauze soaked in xylocaine naphazoline for 20 min. The percentage of XYLOCAINE used is 5%. The 24 mL vial contains 1.2 g of XYLOCAINE and 4.8 mg of NAPHAZOLINE. Topical anesthesia is preferred to a small injection into the plica nasi, so that the anatomy of the plica nasi is not altered and the opening of the ULC-septum angle can be observed accurately. In addition, the most cephalic part of the nasal cavity would not be properly anesthetized and the patient would feel the upward progression of the cannula.

**Fig. 1:**
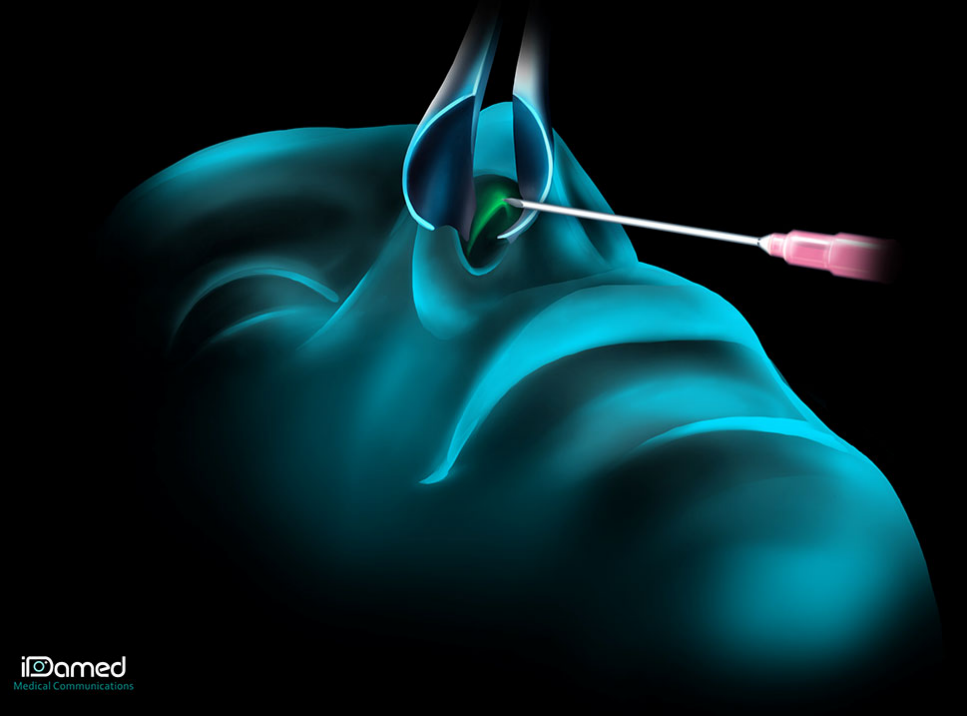
Injection technique: First, a trocar was inserted into the plica nasi

**Fig. 2:**
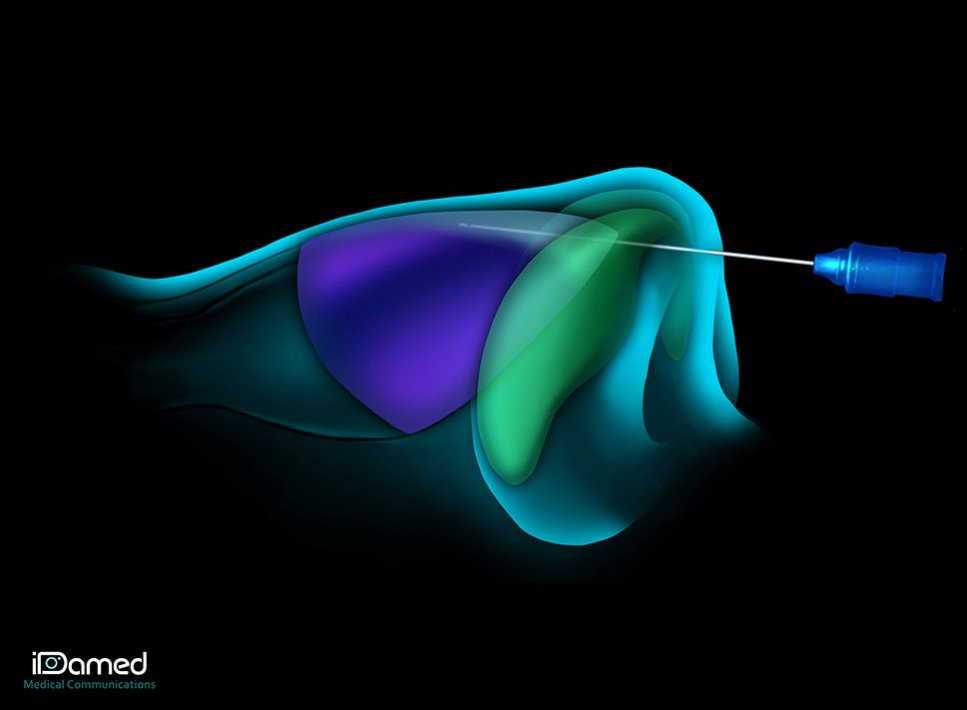
Injection technique: Then, the hyaluronic acid was injected along the ULC-septum angle

Hyaluronic acid (Vivacy® Stylage XXL) was then injected by a senior nasal surgeon along the ULC-septum angle, bilaterally. A trocar was inserted into the plica nasi (Fig. 1) and the HA (0.2–0.5 mL per side, 0.4–1 mL per patient) was injected with a 25G/40 mm cannula along the ULC-septum angle (Fig. 2). The upper limit of the injection is the same as the upper limit of a real cartilage spreader graft: the keystone area. To limit the risk of embolism and skin necrosis, the injection was made using a cannula instead of a needle, and was performed in a submucosal plane, right under the cartilage, which is supposed to be an avascular plane. The progression of the cannula can be seen and/or felt up to the keystone area, and then the injection is retro-traced caudally to the entry point corresponding to the plica nasi. The quantity of HA to be injected was determined by two factors: the severity of the IVC and the importance of the aesthetic deformation of the middle third of the nose. The quantity of HA injected had to be sufficient to cause abduction of the upper lateral cartilages (opening of the ULC-septum angle visualized endonasally), to correct the aesthetic deformity (restoration of the dorsal aesthetic lines), without however causing a disgraceful widening of the middle third. In addition, we were guided by the patient’s feelings. Since, the patient was conscious throughout the procedure, he reported respiratory improvement as soon as the amount of acid injected was sufficient. On top of that, the ULC-septum angle is a very small space, where only a limited quantity of HA can be injected. You can sometimes observe a leak of HA at the entry point in the plica nasi when this space is fully filled with HA (cf Video 1). As the hyaluronic acid plays a role of spreader graft, it must have rheological properties allowing it to oppose the inspiratory collapse of the upper lateral cartilages. That is why we chose a highly cross-linked hyaluronic acid. Adverse effects (bleeding, allergic reaction, skin pain) were recorded. Photographs of the patient were taken before and after HA injection. The injection technique is shown in Video 1.

### Statistical Analysis

Twenty subjects were estimated to be sufficient to reveal a 40-percentage-point reduction in the proportion of patients with IVC after HA injection with sufficient power (alpha risk 5%). Internal valve collapse was defined as a hysteresis loop area greater than 17.3 Pa L s^-1^ in 4-phase rhinomanometry data. The proportions of patients with IVC before and at the two follow-up visits after HA injection (D30 and M12) were compared using McNemar’s test. The before–after comparisons for the secondary variables of interest (NOSE and VAS scores) were performed using paired Wilcoxon tests at D30 and M12. All analyses were performed using R v.4.1.2 (R Foundation for Statistical Computing, Vienna, Austria, www.r-project.org). Differences were considered statistically significant at p < 0.05.

## Results

### Population

Twenty-two patients consulted for nasal obstruction due to post (septo)rhinoplasty IVC between January and April 2023 in the study center and were included in the study. None of the patients had been operated on by the senior surgeon who performed the injections (AM). Patient characteristics are shown in Table 1. The average time (± standard deviation), since (septo)rhinoplasty was nine (± 9) years. The patients all had Caucasian-shaped noses. The middle third of the nose was thin in 55% (12/22) of patients, and 73% (16/22) had an “inverted V” deformity. The mean follow-up was 1.2 (± 0.2) years. At the second follow-up visit (M12), 68% (15/22 patients) were recorded because of loss to follow-up.

**Table 1:** Study population

		n =22
Age (years)		41 (±14)
Female Gender		13/22 (59%)
Height (cm)		169 (±10)
Weight (kg)		64 (±13)
Tobacco use		8/22 (36%)
Atopy		12/22 (55%)
Years since rhinoplasty		9 (±9)
Nasal ethnic type		
Caucasian		22/22 (100%)
African		0/22 (0%)
Asian		0/22 (0%)
Width of the middle third		
Narrow		12/22 (55%)
Normal		10/22 (45%)
Frontal nasal view		
Inverted V deformity		16/22 (73%)
Deviation		3/22 (14%)
Normal		3/22 (14%)
Profile nasal view		
Kyphosis		5/22 (23%)
Saddle		3/22 (14%)
Straight		14/22 (63%)
Septal deviation		0/22 (0%)
Inferior turbinate hypertrophy		0/22 (0%)

The values correspond to the numbers (proportions) for the categorical variables and the means (standard deviation) for the quantitative variables.

### Hyaluronic Acid Injections

**Fig. 3:**
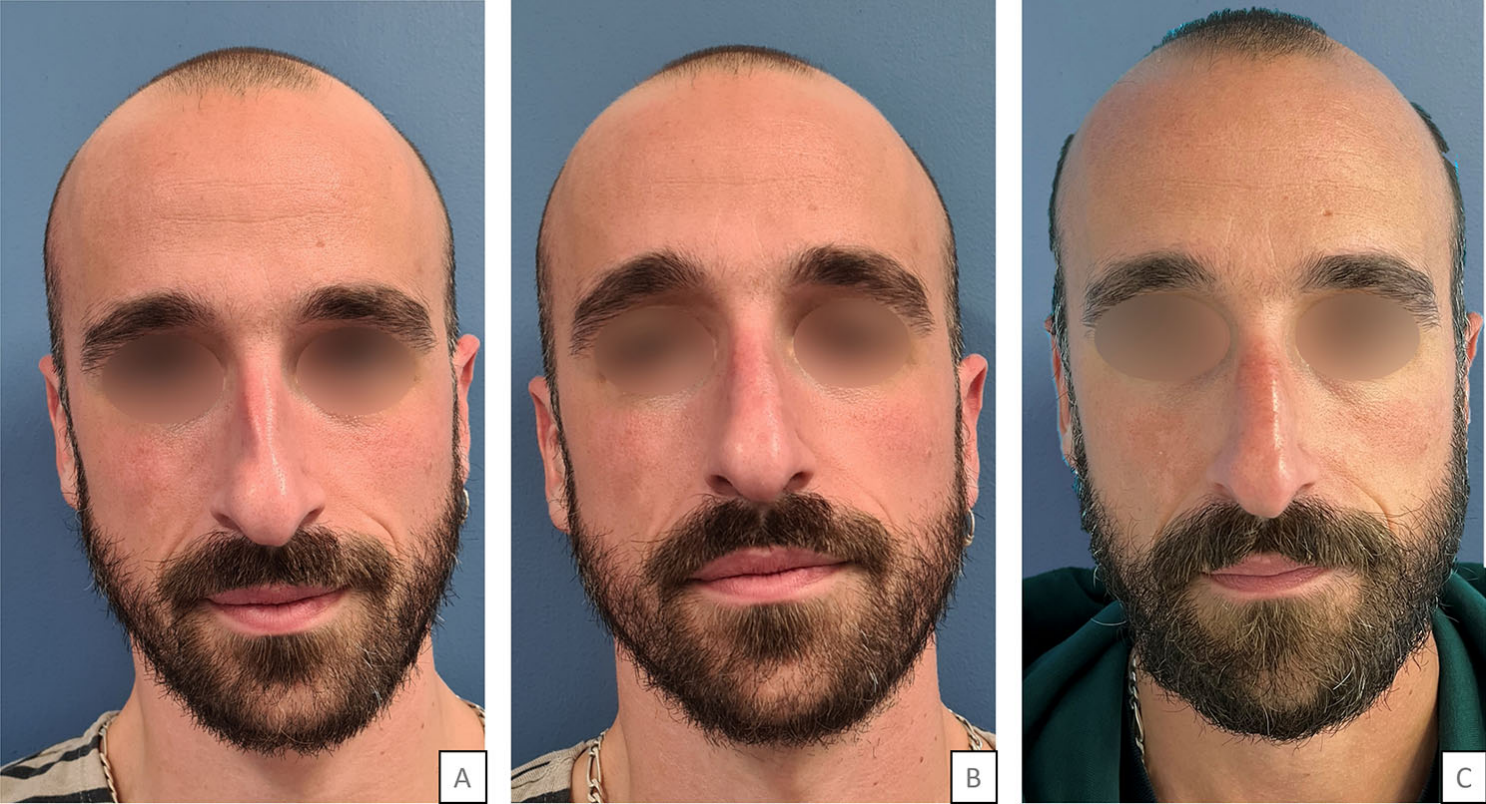
34 years old man who underwent aesthetic rhinoplasty in 2015, with a collapse middle third. 0.7 mL were injected in the right ULC-septum angle, 0.5 mL were injected in the left ULC-septum angle Legend: A = before injection; B = five minutes after injection; C = one month after injection

**Fig. 4:**
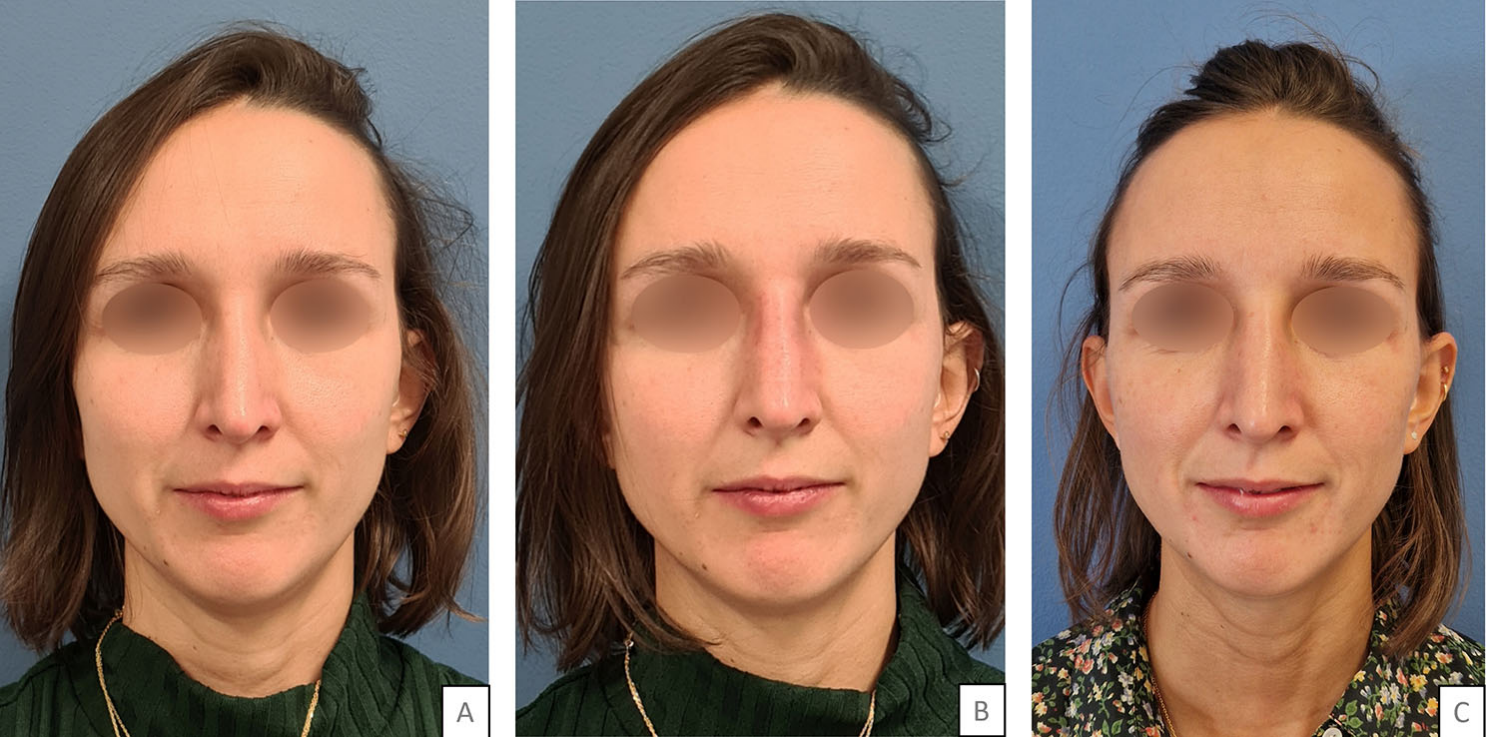
32 years old woman who underwent aesthetic rhinoplasty in 2017, with an “inverted-V” deformity. 0.4 mL were injected in each ULC-septum angle. Legend: A = before injection; B = five minutes after injection; C = one month after injection

The volume of HA injected ranged from 0.2 to 0.7 mL per side, with an average of 0.45 (± 0.10) mL per side. No postinjection adverse effects (bleeding, allergic reaction, skin pain) were recorded. Figures 3, 4 show before-and-after photographs of three patients.

### 4-Phase Rhinomanometry

**Fig. 5:**
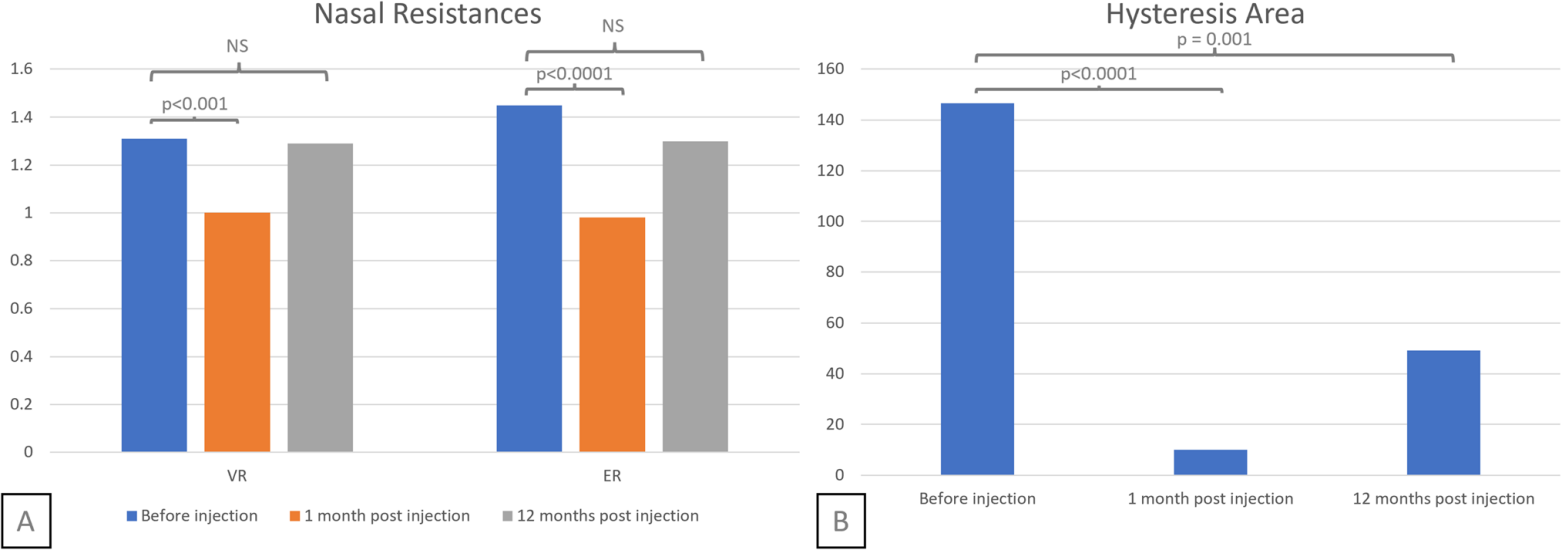
4-phase rhinomanometry results Legend: Nasal Resistances **A** and Hysteresis Area **B**, before injection, one and twelvemonths after. Abbreviations: VR, Vertex Resistance; ER, Effective Resistance

The mean ER decreased from 1.45 (± 0.34) Pa mL s^−1^ before injection to 0.98 (± 0.46) Pa mL s^−1^ at D30 (p < 0.0001), and then increased to 1.30 (± 0.58) Pa mL s^−1^ at M12 (p = 0.06 ; Fig. 5A). The mean VR decreased from 1.31 (± 0.32) Pa mL s^−1^ to 1.00 (± 0.46) Pa mL s^−1^ at 30 days’ follow-up (p < 0.001) and then increased to 1.29 (± 0.56) Pa mL s^−1^ at M12 (p = 0.91 ; Fig. 5A). The mean inspiratory hysteresis area decreased from 147 (± 121) Pa L s^-1^ before injection to 10.0 (± 7.6) Pa L s^-1^ at 30 days’ follow-up (p < 0.0001), and then increased to 49.2 (± 65,9) Pa L s^-1^ at M12 (p = 0.001 ; Fig. 5B). Twenty of the 22 patients (91%) had an inspiratory loop area below the diagnostic threshold for IVC 30 days after injection. At twelvemonths post injection, 8 of the 15 patients (53%) had an inspiratory loop area below the diagnostic threshold for IVC.

### NOSE and VAS Scales

**Fig. 6:**
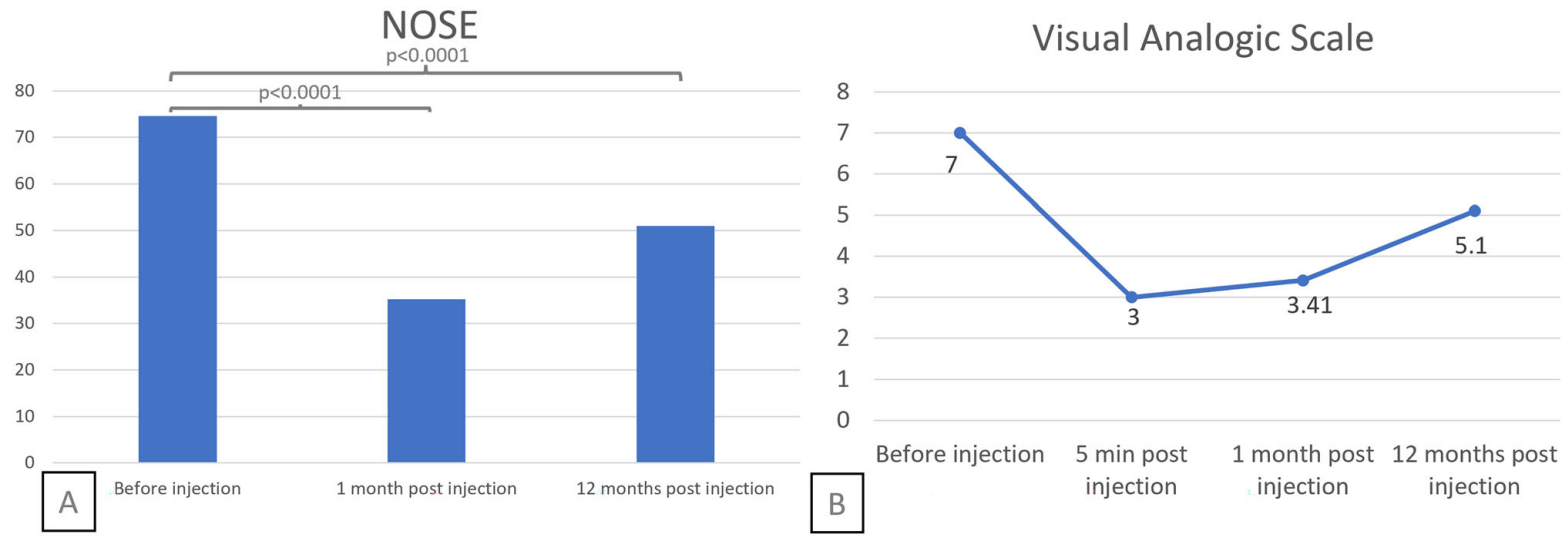
Nasal Obstruction Questionnaires Legend: Mean value of the Nasal Obstruction Symptoms Evaluation (NOSE) scale **A** and Visual Analogic Scale **B**, before injection **A, B**, five minutes after injection (B), at one and twelvemonths postinjection **A, B**

The mean NOSE score decreased from 74.5/100 (± 18.0) before injection to 35.2/100 (± 23.3) at D30, a mean difference of 39.3 points (Fig. 6A). Then, it increased to 51.2/100 (± 24.4) at M12. The mean VAS decreased from 7/10 (± 1.4) before injection to 3/10 (± 1.3) immediately after injection (p < 0.0001), and to 3.4/10 (±1.9) at D30 (p < 0.0001). It increased to 5.1/10 (± 2.8) at M12 (p = 0.001 ; Fig. 6B). The difference between the immediate and D30 improvements in VAS was not significant (p = 0.24).

## Discussion

Hyaluronic acid injection into the internal nasal valve (along the ULC-septum angle) alleviated IVC at 30 days’ follow-up in 20/22 patients consulting for nasal obstruction due to post (septo)rhinoplasty IVC. At twelvemonths’ post injection, results were similar for 8/15 patients assessed. Subjective measures of nasal obstruction (NOSE and VAS) were significantly improved 30 days and twelvemonths after injection. Nevertheless, the technique requires a certain amount of practice to acquire and its success relies on the use of HA with similar characteristics (cross-linking, cohesiveness) to the product used here.

This technique is significantly less costly than surgery (21, 29) and much faster, requiring no more than 25 min (21) to perform (including local anesthesia). The fact that this technique can be performed under local anesthesia, in everyday clinical practice, thus without the risks, time and costs associated with general anesthesia and hospitalization, are considerable advantages. The effects of the procedure are also immediate, while improvements are only observed after three to six months for surgical rhinoplasty (30–32). An additional benefit of these HA injections is that they correct “inverted V” deformities, present in 73% (16/22) of the patients in our study.

The drawbacks of this procedure include the risk of discomfort for some patients, particularly those with significant fibrosis in the nasal valve, as this may hinder the passage of the cannula. Patients should also be warned of the risk of a slight widening of the middle third of the nose (24, 33, 34), which can however be beneficial when this is too thin. It is essential in our view that the HA be injected with a cannula rather than a needle (35) because the blunt tip of the cannula limits the risk of vascular puncture and therefore of embolism. Although the risk is very low [0.5%(36, 37)], embolism can lead to serious adverse effects such as skin necrosis [3/5000 patients in Harb et al.’s review (36)], blindness or stroke. Surgeons should also bear in mind that the nasal anatomy may have been altered by the initial surgery. Injection into a nose that has already been operated on presents a greater embolic risk. The scar and fibrotic tissue (38) present in the nose after rhinoplasty is less well vascularized and therefore more at risk of necrosis. In addition, the vascular anatomy may be modified and disrupt the classical injection markers. Finally, risk factors such as diabetes, active smoking, vascular pathology, and a delay after primary rhinoplasty of less than one year must be considered (39). Nevertheless, the injection is performed in a submucosal plane, right under the cartilage, which is supposed to be an avascular plane. It’s not the same injection plane as an aesthetic non-surgical rhinoplasty. Thus, the risk can be considered as limited. The final disadvantage of this approach is that HA resorbs over time. The duration of efficacy in the nose is not well known but seems to be longer than in other areas of the face (36). This result is consistant with ours, and is possibly due to less frequent muscle contractions in the nasal valve area and by the presence of postoperative fibrosis.

There are few ways to objectively diagnose IVC (40). Patel et al. proposed a method based on the visibility of the middle turbinate (41) but this classification only considers the static component of the valve and does not perfectly reflect its pathophysiology. Tsao et al. proposed another approach based on a dynamic study of the internal valve (42), but this is subject to measurement bias. We chose 4-phase rhinomanometry because this technique correlates particularly well with patients’ perceptions and has been shown to have excellent sensitivity and specificity (respectively 88.3 ; 89.9%) for the objective diagnosis of IVC (6).

Based on a meta-analysis of 31 articles, Rhee et al. (28) defined a 30-point improvement in the NOSE score and a 3.0-point improvement in VAS as criteria for surgical success. The average improvements reported here (39.3 points for the NOSE and 3.4 points for the VAS score) are therefore clinically significant and were observed immediately after injection. The assessment of nasal obstruction immediately after the procedure may have been biased by the application of xylocaine naphazoline, which has a vasoconstrictive effect on the nasal mucosa, but the subjective improvements were maintained 30 days after injection. These results suggest that the effects of this technique are felt immediately and last for several months.

This technique, initially described by Nyte (20) in 2007, is routinely used by plastic surgeons based on expert knowledge and experience, but the present results are the rigorous evidence of its efficacy. To our knowledge, only one study (43) assessed the effectiveness of HA injection in the nasal cavity as a treatment for IVC. Although the diagnostic method used to assess IVC differed slightly, our results confirm an objective improvement. Our study group was homogeneous and representative of the target population (patients with post-(septo)rhinoplasty IVC). The limitations of this study include its small size (n=22) and the absence of a control group. Furthermore, although (septo)rhinoplasty is the most frequent cause of IVC (8), whether similar improvements are achieved for other etiologies remains to be investigated.

## Conclusion

In conclusion, the results of this study support the use of HA injection into the internal nasal valve (along the ULC-septum angle) as a treatment for the post-(septo)rhinoplasty IVC as one-year follow-up data remains quite satisfactory regardless of the resorption (47% of patients).

## Supplementary Information

Below is the link to the electronic supplementary material.

Supplementary file1 (MP4 28642 KB)
